# Using Advanced Cell Culture Techniques to Differentiate Pluripotent Stem Cells and Recreate Tissue Structures Representative of Teratoma Xenografts

**DOI:** 10.3389/fcell.2021.667246

**Published:** 2021-05-06

**Authors:** L. A. Smith, A. Hidalgo Aguilar, D. D. G. Owens, R. H. Quelch, E. Knight, S. A. Przyborski

**Affiliations:** ^1^Department of Biosciences, Durham University, Durham, United Kingdom; ^2^Reprocell Europe, NETPark, Sedgefield, United Kingdom

**Keywords:** pluripotent stem cells, embryoid bodies, three-dimensional cell culture, porous scaffold membrane, tissue differentiation, teratoma assay, *in vitro*

## Abstract

Various methods are currently used to investigate human tissue differentiation, including human embryo culture and studies utilising pluripotent stem cells (PSCs) such as *in vitro* embryoid body formation and *in vivo* teratoma assays. Each method has its own distinct advantages, yet many are limited due to being unable to achieve the complexity and maturity of tissue structures observed in the developed human. The teratoma xenograft assay allows maturation of more complex tissue derivatives, but this method has ethical issues surrounding animal usage and significant protocol variation. In this study, we have combined three-dimensional (3D) *in vitro* cell technologies including the common technique of embryoid body (EB) formation with a novel porous scaffold membrane, in order to prolong cell viability and extend the differentiation of PSC derived EBs. This approach enables the formation of more complex morphologically identifiable 3D tissue structures representative of all three primary germ layers. Preliminary *in vitro* work with the human embryonal carcinoma line TERA2.SP12 demonstrated improved EB viability and enhanced tissue structure formation, comparable to teratocarcinoma xenografts derived *in vivo* from the same cell line. This is thought to be due to reduced diffusion distances as the shape of the spherical EB transforms and flattens, allowing for improved nutritional/oxygen support to the developing structures over extended periods. Further work with EBs derived from murine embryonic stem cells demonstrated that the formation of a wide range of complex, recognisable tissue structures could be achieved within 2–3 weeks of culture. Rudimentary tissue structures from all three germ layers were present, including epidermal, cartilage and epithelial tissues, again, strongly resembling tissue structure of teratoma xenografts of the same cell line. Proof of concept work with EBs derived from the human embryonic stem cell line H9 also showed the ability to form complex tissue structures within this system. This novel yet simple model offers a controllable, reproducible method to achieve complex tissue formation *in vitro*. It has the potential to be used to study human developmental processes, as well as offering an animal free alternative method to the teratoma assay to assess the developmental potential of novel stem cell lines.

## Introduction

The study of human tissue differentiation continues to be of significant interest and importance to a wide range of biological fields, including human developmental biology, stem cell science, and regenerative medicine. Although notable progress is being made, many events and mechanisms during this process remain to be clarified, with a wide range of techniques used to investigate these processes. Both *in vivo* and *in vitro* approaches are employed; each has its own unique advantages and limitations regarding the method and the information that can be elucidated (see Table 1 for brief overview and comparisons). While working with human embryos is the most physiologically relevant and accurate option, work of this nature is challenging due to the logistical issues of obtaining material for use in experiments, the associated ethical concerns and regulatory constraints on culture practices (Cavaliere, 2017). Animal models to study tissue differentiation offer a feasible, more accessible solution enabling long term *in vivo* studies. Moreover, general developmental processes permit the transposition of data obtained from animal studies to human differentiation events, for example, the identification of general mechanisms, such as the formation of the body axis. However, the use of animals is more indicative than conclusive due to heterogeneity in many aspects of the developmental process, including blastocyst formation and organogenesis (Irie and Kuratani, 2014; Rossant, 2015). In addition, there is a commitment to decrease the use of animal based models in favour of reducing, refining and replacing these experiments with appropriate and sustainable alternatives (Festing and Wilkinson, 2007).

**TABLE 1:** Strategies used to study the development and differentiation of human cells and tissues–advantages and limitations.

	Human embryo work	Surrogate animal models	Conventional 2D cell culture	Embryoid body formation	Teratoma assay
**Source**	Human tissue	Animal tissue	Cell lines Primary cells Pluripotent stem cells	Pluripotent stem cells	Pluripotent stem cells
**Advantages**	Natural, normal human tissue development Physiologically relevant	Complex tissue differentiation Ability to study events in depth as they occur Fewer regulatory issues and restrictions	Simple directed differentiation Reductionist view enables elucidation of pathways and mechanisms Good reproducibility	Capable of recapitulating events of early embryogenesis Some physiological similarity of basic early processes	Complex tissue differentiation Evidence of distinct tissue formation Demonstration of pluripotency
**Limitations**	Difficulties obtaining materials Highly regulated field Ethical issues surrounding embryo work Specific training required Donor variability	Data not truly representative of human process Mechanisms do not always extrapolate to man Ethical issues surrounding animal usage Laborious and can take significant time	Cells adapted to a non-physiological environment Responses and function may not be representative of *in vivo* due to reductionist view 3D complex tissue development not possible	Can suffer from limited viability Necrotic core can be an issue Mainly epithelial organoid models Variable reproducibility with some methods	Ethical issues surrounding animal usage Protocols laborious and highly variable between labs Tumour growth influenced by multiple biological factors Can be difficult to delineate between tumour/host tissue

*A range of methods and techniques are currently used to study the early events of human tissue development and the differentiation of stem cells, with each having various advantages and limitations depending on the type of study being performed. These methods involve the study of either cells or tissues using in vivo and in vitro approaches. Each type of method enables the study of cell and molecular processes at specific times during development in different ways, and offer alternative approaches to follow stem cell differentiation and the formation of tissue derivatives.*

**FIGURE 1:**
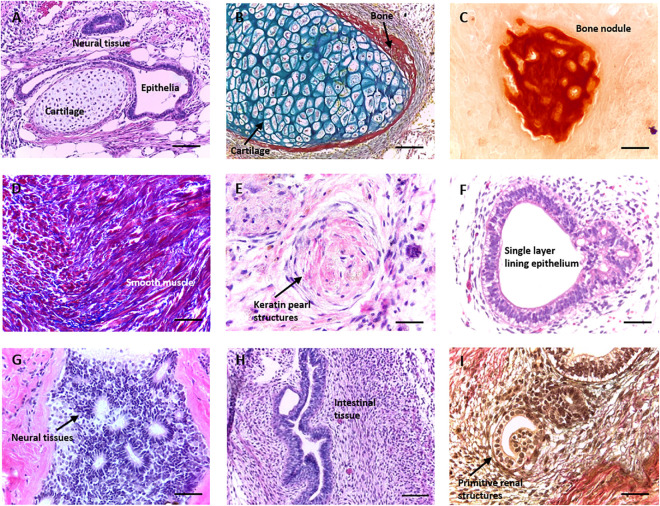
Formation of morphologically identifiable tissue structures in a teratoma derived from human pluripotent stem cells. Histological analysis of teratomas formed from human embryonic stem cell lines (such as the H9 lineage) following implantation into immunodeficient mice. When grown as a xenograft, pluripotent stem cells (PSCs) form a range of complex differentiated tissue structures within a teratoma. The assay is normally used to assess the functional aspect of cellular pluripotency, and the presence of tissues from all three germ layers is indicative of the property. Images are haematoxylin and eosin stained unless otherwise stated. **(A)** Typical section through a teratoma showing multiple germ layer structures including neural tissue (ectoderm), cartilage (mesoderm), and a single layer lining epithelium (endoderm). **(B)** Cartilage (blue) with some peripheral bone (red) stained using Weighert’s stain. **(C)** Bone nodule stained using alizarin red. **(D)** Smooth muscle tissue (red) and surrounding ECM (blue) stained using Masson’s trichrome. **(E)** Primitive keratin pearl structure. **(F)** Single layer epithelia lined lumen. **(G)** Neural tissues with a number of neural rosettes. **(H)** Intestinal epithelial structures. **(I)** Primitive renal structures stained using Weighert’s stain. Representative structures shown. Scale bars: **(A–D)** 100 μm and **(E–I)** 50 μm.

The teratoma xenograft assay, a method primarily employed for assessing the pluripotency of novel pluripotent stem cell (PSC) lines, can also be used to study the process of human tissue development and formation (Aleckovic and Simón, 2008; Jamil et al., 2013; Hultman et al., 2014; Al-Jomard and Amarin, 2017). This technique involves the implantation of PSCs into an immune deficient host to form a solid xenograft tumour over several weeks. It remains the definitive test in which highly complex, recognisable, mature tissue structures derived from the three primary germ layers can be formed using human PSC populations (Figure 1). Tissues of this complexity have not been reproduced to the same degree *in vitro*. However, there are a number of limitations to the teratoma assay that prevent its widespread adoption, notably: use of an animal host and ethical/regulatory issues, protocol variability, prolonged time scale, assay expense, variable reporting, and lack of quantifiable data (Müller et al., 2010; Gropp et al., 2012; Buta et al., 2013; Bouma et al., 2017).

*In vitro* studies involving PSC lines are of interest due to their inherent reproducibility, as well as the ability to direct their differentiation towards specific cell types. In the past, human PSC studies have relied on culturing cells in a traditional manner on flat glass or plastic surfaces and such research has contributed significantly to the knowledge base regarding the differentiation of human tissues (Schuldiner et al., 2000). While inexpensive, homogenous and simple, these methods do not represent the *in vivo* microenvironment, resulting in the exposure of cells to artificial cues and leading to the adoption of an abnormal morphology and function (Cukierman et al., 2002; Baker and Chen, 2012).

Under the appropriate growth conditions, PSCs placed in close contact can self-organise to form spherical structures known as embryoid bodies (EBs), that can recapitulate some of the early aspects of human development and can be used as a basic method to assess aspects of pluripotency in new human PSC lines (Meng et al., 2014). EB formation is often used as a starting point for more complex differentiation protocols. However, this method also has significant limitations, principally surrounding the increasing diffusion distances between differentiating cells and the culture medium as EBs grow in size. Multiple studies report the formation of a central core of cell death as EB size increases, in both spontaneously differentiated and lineage directed EBs (Dang et al., 2002; Gothard et al., 2009; Son et al., 2011; Van Winkle et al., 2012; Giandomenico et al., 2019).

The development of a range of three dimensional (3D) culture technologies and their established widespread use provides significant opportunities for enhanced *in vitro* models (Cukierman et al., 2002; Yamada and Cukierman, 2007; Baker and Chen, 2012). These methods provide not only more physiologically relevant chemical and physical cues which more accurately recapitulate the nuances of the *in vivo* microenvironment, but can also allow for improved nutritional support of cells and nascent tissue structures. This enhanced support in turn permits prolonged culture, providing sufficient time for the differentiation of cells and formation of complex, mature, functional tissue constructs. The combination of new culture technologies with existing techniques is leading to new approaches to enhance cell growth such as slice culture of cerebral organoids on permeable membranes as opposed to the seeding of entire neurospheres onto 3D scaffolds. Studies such as these have ultimately aimed to reduce the diffusion distances in these developing tissue structures, noting subsequent improvements in viability (Giandomenico et al., 2019), and changes in differentiation (Nestor et al., 2013).

In this study, we have utilised the well-established technique of EB formation in conjunction with membrane technology to develop a novel model to facilitate tissue development using spontaneously differentiating human PSC derived EBs. Through seeding EBs onto inert, porous polystyrene scaffold membranes, we have shown that the viability of EB-derived structures can be improved, allowing for their prolonged maintenance on the membrane and the formation of various complex tissue structures representative of the three primary germ layers not normally observed in the EB. Initial work with human pluripotent embryonal carcinoma (EC) stem cells was performed to test and optimise the experimental approach prior to moving onto work using embryonic stem cells. Crucially, the tissue structures formed using this new approach were comparable to xenograft teratoma tissues derived from the same cell line *in vivo*. We demonstrate that this novel *in vitro* system provides a controllable and robust method to study and direct the formation of rudimentary human tissues over time, as well as providing a potential animal free alternative to the traditional teratoma assay for the assessment of stem cell pluripotency.

## Materials and Methods

### Routine Maintenance of TERA2.cl.SP12 Human Embryonal Carcinoma Cell Line

TERA2.cl.SP12 EC cells [previously isolated in Przyborski (2001)] were maintained at high confluency in a complete medium consisting of Dulbecco’s Modified Eagles Medium with high glucose (DMEM-HG, Gibco, Invitrogen, Paisley, United Kindom), 10% heat treated Foetal Bovine Serum (FBS, Gibco), 2 mM L-glutamine (Gibco) and 100 active units/mL of Penicillin/Streptomycin (Gibco) at 37°C, 5% CO_2_, 95% air. Cells were passaged at 100% confluency by rolling acid washed glass beads across the surface of the flask to dislodge cells and subsequently split at a ratio of 1–3 into new T25 cm^2^ flasks (BD Falcon, Erembodegem, Belgium).

### Formation, Maintenance and Differentiation of TERA2.cl.SP12 Embryoid Bodies

TERA2.cl.SP12 EC cells at full confluency were treated with 0.25% trypsin/EDTA to achieve a single cell suspension and counted using the Trypan Blue exclusion assay in order to obtain accurate viable cell numbers. 1.5 × 10^6^ cells were seeded per sterile, non-tissue-culture treated 90 mm Petri dish (Fisher Scientific UK, Loughborough, United Kingdom), to form EBs and differentiate for up to 14 days. Media was changed every 2–3 days by transferring the EBs to a 50 mL Falcon tube, allowing them to settle and aspirating off used media. Fresh media was added and the suspension placed back into a new 90 mm sterile non-tissue-culture treated Petri dish.

### Routine Maintenance of CGR8 Murine Embryonic Stem Cell Line

CGR8 murine embryonic stem cells were maintained as advised on the European Collection of Authenticated Cell Cultures (ECACC) website (Public Health England, 2021). Briefly, T25 cm^2^ flasks (Nunc) were coated with a 0.2% gelatin solution prior to use, and cells were maintained in pluripotency maintenance medium consisting of Glasgow Minimum Essential Medium (GMEM, Sigma Aldrich, Dorset, United Kingdom), 10% ES cell qualified Foetal Bovine Serum (qFBS, Gibco), 2 mM L-glutamine, 100 active units/mL Penicillin/Streptomycin, 1,000 active units/mL LIF (Amsbio, United Kingdom) and 0.05 mM β-mercaptoethanol (Gibco). Cells were passaged on reaching 70–80% confluency using 0.25% trypsin/EDTA, and splitting at a ratio of 1–10. Cells were incubated at 37°C, 5% CO_2_, 95% air.

### Formation and Maintenance of CGR8 Embryoid Bodies

Prior to forming EBs using the CGR8 cell line, Aggrewell 800^TM^ (Stem Cell Technologies, Cambridge, United Kingdom) plates were prepared for use as per the manufacturer’s instructions. Briefly, plates were washed in culture medium and centrifuged at high speed to displace air bubbles in the base of the microwells. Cells at 70–80% confluency were treated with 0.25% trypsin/EDTA to achieve a single cell suspension and counted using the Trypan Blue exclusion assay. 3 × 10^5^ cells were seeded per well to form 300 EBs of around 1,000 cells. Cells were allowed to form EBs over 3 days with half media changes each day, before being transferred to a 90 mm sterile, untreated Petri dish for a further 5 days.

### Differentiation of CGR8 Embryoid Bodies Using Morphogens

Exogenous morphogens were added to CGR8 ES cells during Aggrewell culture in order to direct the differentiation of the EBs towards specific primary germ layers. The following morphogens were used: Dorsomorphin (Sigma Aldrich), activin receptor-like kinase inhibitor SB431542 (Sigma Aldrich), basic fibroblast growth factor (*E. Coli* expression system, bFGF, Fisher Scientific UK), Activin A (HEK293 cell expression system, Fisher Scientific UK) and bone morphogenetic protein 4 (HEK293 cell expression system, BMP4, Fisher Scientific UK). Morphogens and concentrations were taken from the recent International Stem Cell Initiative Study (Allison et al., 2018).

### Routine Maintenance of H9 Human Embryonic Stem Cell Line

H9 human embryonic stem cells (hESCs, WiCell) and maintained in feeder free conditions in six well plates coated with Matrigel hESC qualified Matrix (Corning, Flintshire, United Kingdom), prepared and diluted according to the manufacturer’s instructions. Cells were maintained in mTESR Plus media (Stem Cell Technologies) prepared and stored as per the manufacturer’s instructions, with media changes every day and incubated at 37°C, 5% CO_2_, 95% air. Cells at 70–80% confluency were passaged via an enzyme free method using ReLeSR (Stem Cell Technologies) to detach cells and split into new wells at a ratio of 1–6.

### Formation and Maintenance of H9 Embryoid Bodies

Prior to the creation of EBs, Aggrewell plates were prepared according to the manufacturer’s instructions, as described above. Rho kinase (ROCK) inhibitor Y27632 (Tocris Biosciences, Abingdon, United Kingdom) was added to cells intended for EB creation at a final concentration of 10 μM for 60 min prior to use, as well as to media during counting and EB formation. To create EBs, H9 cells at 70–80% confluency were detached using ReLeSR, gently pipetted to form a single cell suspension and counted using the Trypan Blue exclusion assay. Differentiation medium consisted of Knock Out DMEM (Fisher Scientific UK), 20% Knock Out Serum Replacement (Fisher Scientific UK), 1 mM L-glutamine, 0.1 mM non-essential amino acids (Sigma Aldrich), 0.1 μM β-mercaptoethanol and 10 μM Y27632. 3 × 10^6^ cells were seeded per well to form 300 EBs of around 10,000 cells. EBs were maintained in Aggrewells for 3 days with half media changes, during which the ROCK inhibitor was removed. EBs were then transferred to six well flat-bottom non-treated plates (Stem Cell Technologies) washed with Anti-Adherence Rising Solution (Stem Cell Technologies, United Kingdom) to prevent EB aggregation and maintained for a further 5 days.

### Preparation of Alvetex^®^ Membranes and Seeding and Maintenance of EBs

Prior to use, six-well inserts containing Alvetex^®^ Polaris polystyrene membranes (Reprocell Europe, United Kingdom) were treated using previously established protocols. Briefly, inserts were washed in 70% ethanol, before being rinsed in sterile phosphate buffered saline (PBS) twice, and placed in a six-well plate (Greiner Bio-One, Stonehouse, United Kingdom) with the appropriate medium.

After formation, PSC derived EBs were transferred individually to the six-well inserts, with around 6–8 being placed on each insert in submerged culture. For H9 EBs, Alvetex^®^ inserts were coated in Matrigel Growth Factor Reduced Basement Membrane Matrix prior to seeding to aid attachment. EBs were then cultured for a defined timescale, before washing in PBS and fixation in 4% paraformaldehyde (PFA, Fisher Scientific) overnight at 4°C.

### Sample Processing and Sectioning

Fixed inserts were washed in PBS before dehydration in 30% ethanol, and then 50% ethanol. Inserts were stained with 0.1% crystal violet in 70% ethanol in order to visualise the 3D cultures more easily during the embedding and sectioning process. Inserts were then dehydrated through a series of ethanols: 80, 90, 95, and 100%. Samples were incubated in Histoclear I (Scientific Laboratory Supplies, SLS, Nottingham, United Kingdom) for 20 min, before transferring to a Histoclear I:wax (Fisher Scientific) 50:50 solution at 60°C for 30 min. Samples were then placed in 100% molten wax at 60°C and incubated for 1 h. Inserts were embedded in wax using embedding moulds and orientated appropriately to allow for transverse or longitudinal sectioning parallel to the plane of the membrane. EBs were processed in a similar manner; the process was performed in a 15 mL Falcon tube (Fisher Scientific), with EBs allowed to settle to the base before solutions were changed. Samples were sectioned at 6 μm on a Leica RM2125 RT Microtome and mounted on SuperFrost charged slides (Fisher Scientific).

### Haematoxylin and Eosin Staining

For haematoxylin and eosin staining, slides were deparaffinised in Histoclear I and rehydrated, finishing in distilled water (dH_2_O). Slides were incubated in Mayer’s haematoxylin (Sigma Aldrich) for 5 min, and rinsed in distilled water for 30 s. Slides were washed in alkaline alcohol for 30 s to blue the nuclei, before being placed in 95% alcohol for 30 s. Slides were incubated in eosin dissolved in 95% ethanol for 1 min, before washing and dehydration in 95 and 100% ethanol. Finally, slides were further dehydrated and cleared twice in Histoclear I for 5 min each before mounting using Omnimount (SLS).

### Masson’s Trichrome Staining

Samples were stained with Masson’s trichrome to allow for the identification of specific tissue identities, such as extracellular matrix rich areas including cartilage and bone, muscle tissues and tissues containing keratin. To stain, slides were deparaffinised in Histoclear I and rehydrated through a series of ethanols, finishing in dH_2_O. Slides were stained in Weighert’s iron haematoxylin solution for 15 min or until nuclei were stained black, before washing in running tap water for 10 min. Slides were rinsed in dH_2_O, before staining in biebrich scarlet/acid fuschin solution for 8–10 min. Slides were rinsed in dH_2_O, before incubating in phosphotungstic/phosphomolybdic acid for 12 min. Slides were rinsed in dH_2_O before staining in aniline blue for 7 min, and washing in dH_2_O again. Slides were incubated in 1% acetic acid to remove excess stain, washed a final time in dH_2_O before dehydration back through a series of ethanols. Slides were cleared twice in Histoclear I for 5 min each before mounting in Omnimount.

### Weighert’s Staining

Weighert’s stain was also used to better identify tissue structures within samples, in particular to differentiate specifically between cartilage and bone tissues. Slides were deparaffinised in Histoclear I and rehydrated through ethanols, finishing in dH_2_O. Slides were stained in alcian blue for 5 min, before washing in dH_2_O. Slides were then stained in Weighert’s iron haematoxylin for 20 min, before rinsing in running tap water for 10 min. Slides were washed in dH_2_O, before dehydrating in 50% ethanol and incubating in 1% acid alcohol. Slides were then dehydrated in 70% ethanol, washed in dH_2_O and stained in Ponceau S solution for 10 s. Slides were dehydrated through ethanols and cleared twice Histoclear I for 5 min each, before mounting in Omnimount.

### Alizarin Red Staining

Alizarin red staining was used in order to identify the rudimentary bone tissues within samples. Slides were deparaffinised in Histoclear I and rehydrated through ethanols, finishing in dH_2_O. Slides were then stained in alizarin red solution (pH 4.1–4.3) for 2 min, before excess stain was blotted from the slides. Slides were dipped in acetone 20 times to dehydrate, before dipping in a 50:50 acetone: Histoclear I solution 20 times. Slides were cleared twice in Histoclear I for 3 min each, before mounting in Omnimount.

### Immunofluorescent Staining

Slides were first deparaffinised in Histoclear I for 15 min before being rehydrated for 5 min each in 100% ethanol, 95% ethanol, 70% ethanol, and dH_2_O. Antigen retrieval was performed by incubating slides in citrate buffer (pH 6) at 95°C for 20 min. After slides had cooled, samples were blocked in a blocking buffer consisting of either 20% normal goat serum (NGS, Sigma Aldrich) or, for CGR8 teratoma slides, 20% newborn calf serum (NCS, Fisher Scientific UK) in 0.4% Triton-X-100 in PBS. Slides were blocked for 1 h at room temperature, and primary antibodies diluted in blocking buffer were added at the appropriate concentrations, and incubated at 4°C overnight. Primary antibodies used were as described in Table 2. The following day, slides were washed three times in PBS before fluorescently conjugated secondary antibodies and the nuclear stain Hoechst diluted in blocking buffer were added to the slides, as described in Table 3. Slides were incubated at room temperature for 1 h before washing three times in PBS. Slides were mounted in Vectashield^TM^ (Vector Labs, Peterborough, United Kingdom) and a cover slip placed on top, which was sealed around the edges using nail varnish. Slides were stored at 4°C until imaging using a Zeiss LSM 880 confocal microscope with Airyscan.

**TABLE 2:** Primary antibodies used to identify cell and tissue phenotypes during immunofluorescent staining.

Primary antibody	Species	Dilution	Product code	Supplier
Class III β tubulin	Rabbit	1:600	#802001	Biolegend
Nestin	Mouse	1:200	MAB5326	Merck Millipore
Oct 4	Rabbit	1:100	ab19857	Abcam
Cytokeratin 8	Mouse	1:200	C5301	Sigma Aldrich
Cytokeratin 10	Rabbit	1:100	ab76318	Abcam
α smooth muscle actin	Mouse	1:100	ab7817	Abcam
Fibronectin	Rabbit	1:100	ab23750	Abcam
E-cadherin	Mouse	1:200	#610181	BD Biosciences
Type IV collagen	Rabbit	1:100	ab6586	Abcam

**TABLE 3:** Secondary antibodies and nuclear stains used during immunofluorescent staining.

Secondary antibody/stain	Dilution	Product code	Supplier
Goat Anti-Mouse Alexa Fluor 594	1:1000	A11005	Invitrogen, Fisher Scientific UK
Goat Anti-Rabbit Alexa Fluor 488	1:600	A11034	Invitrogen, Fisher Scientific UK
Hoechst 33342	1:1000	H3570	Fisher Scientific UK

### TUNEL Staining

The DeadEnd^TM^ Fluorometric TUNEL assay system (Promega, Southampton, United Kingdom) was used to assess apoptosis in PFA fixed, paraffin embedded samples. The manufacturer’s protocol was followed. Briefly, slides were deparaffinised in Histoclear I twice for at least 5 min per wash, rehydrated in 100% ethanol for 5 min, and further rehydrated in ethanols for 3 min in each of the following: 100, 95, 80, 70, and 50%. Slides were washed in 0.85% NaCl solution for 5 min, washed in PBS for 5 min and fixed in 4% PFA at room temperature for 15 min, before two further washes in PBS for 5 min. Proteinase K solution was added and incubated at room temperature for 8–10 min. Slides were washed in PBS and fixed in 4% PFA again for 5 min at room temperature, before washing in PBS again. A positive control slide was created by adding DNase solution to a slide for 5 min, and washing in dH_2_O. Excess liquid was removed from all slides, and Equilibration buffer added for 5–10 min, during which the reaction mixture was prepared and kept on ice. Equilibration buffer was composed of the following: 200 mM potassium cacodylate (pH 6.6 at 25°C), 25 mM *Tris*-HCl (pH 6.6 at 25°C), 0.2 mM dithiothreitol, 0.25 mg/ml Bovine Serum Albumin and 2.5 mM cobalt chloride. Equilibration buffer was removed and the reaction mixture added to each slide and incubated for 60 min at 37°C in a humidified slide tray. To stop the reaction, slides were placed in 2X SSCC buffer in the dark for 15 min at room temperature. Slides were washed twice in PBS for 5 min, nuclei stained using Hoechst and coverslips mounted in Vectashield^TM^ with nail varnish. Slides were stored at 4°C until imaging using the Zeiss LSM 880 confocal microscope.

### Live Staining Using Caspase 3/7 Stain

To assess apoptotic cell death in live cultures, a caspase 3/7 stain was used. A sample of EBs or 3D cultures were transferred to a sterile 96 well round bottom plate. Nexcelom ViaStain^TM^ Live Caspase 3/7 stain (Nexcelom Bioscience Ltd., Manchester, United Kingdom) was prepared according to the manufacturer’s protocol, diluting the 1 mM stock solution in EB maintenance media to a final concentration of 2 μM. Media was removed from the EBs/3D cultures and 200 μL of the staining media was added to each well to cover the structures in the 96 well plate before incubation overnight at 37°C, 5% CO_2_. The following day, EBs/3D cultures were washed in PBS and stained for 5–10 min in Hoechst solution diluted in PBS. EBs were then washed in PBS again and transferred to ibidi μ dishes (ibidi, Martinsried, Germany) in around 200 μL of PBS to image immediately on the confocal microscope.

### Teratoma Xenograft Assay

Cells were routinely maintained in the culture conditions described above until the assay date. On the day of the assay, cells were detached using the 0.25% trypsin/2 mM EDTA method and counted using the Trypan Blue Exclusion Assay. Cells were adjusted to a density of 5 × 10^5^ cells per 100 μL, and 100 μL of cell suspension was combined with 100 μL of Growth Factor Reduced Matrigel matrix (Corning, Fisher Scientific UK) and loaded into 1 mL syringes with 21G needles which had been cooled in the freezer prior to use. The loaded syringes were kept covered and on ice for transport to the animal house. Hosts were young male NOD SCID Gamma mice, and each host was injected subcutaneously with one loaded syringe into the flank, with four mice injected for each condition giving a potential for four total tumours per condition. Animals were monitored during the growth period, and were weighed on commencing the experiment, prior to injection, and twice weekly to assess tumour growth. Before the tumour reached 5% of the total body weight of the mouse, the animal was sacrificed according to a Schedule 1 method and the tumour was surgically excised. All procedures were completed under licence (PPL60/4508 and PPL60/3579) and permission according to the guidelines of the Home Office, United Kingdom.

## Results

### *In vivo* Teratomas Derived From Human Pluripotent Stem Cells Are Able to Form a Wide Range of Structures Representative of the Three Primary Germ Layers

At present, the xenograft teratoma assay remains the only technique which is able to generate complex, differentiated tissue structures from PSCs. The presence of structures from all three primary germ layers is sufficient to confirm stem cell pluripotency: Figure 1 demonstrates the range and complexity of structures which form within a typical teratoma derived from hESCs. While the overall arrangement of tissue structures is disorganised within the xenograft (A), specific tissue types are clearly identifiable with distinct cellular morphologies and organisation, and their identities may be further validated using histological stains. Examples include ectoderm derived neural tissue and keratin pearls (G and E), mesoderm derived bone, dense connective tissue, cartilage and muscle (B, C, and D) and endoderm derived epithelial structures (F, H, and I).

### Rationale for the Development of a Novel *in vitro* Culture Method to Prolong the Viability of PSC Derived EBs to Enhance the Differentiation of Tissue Derivatives

**FIGURE 2:**
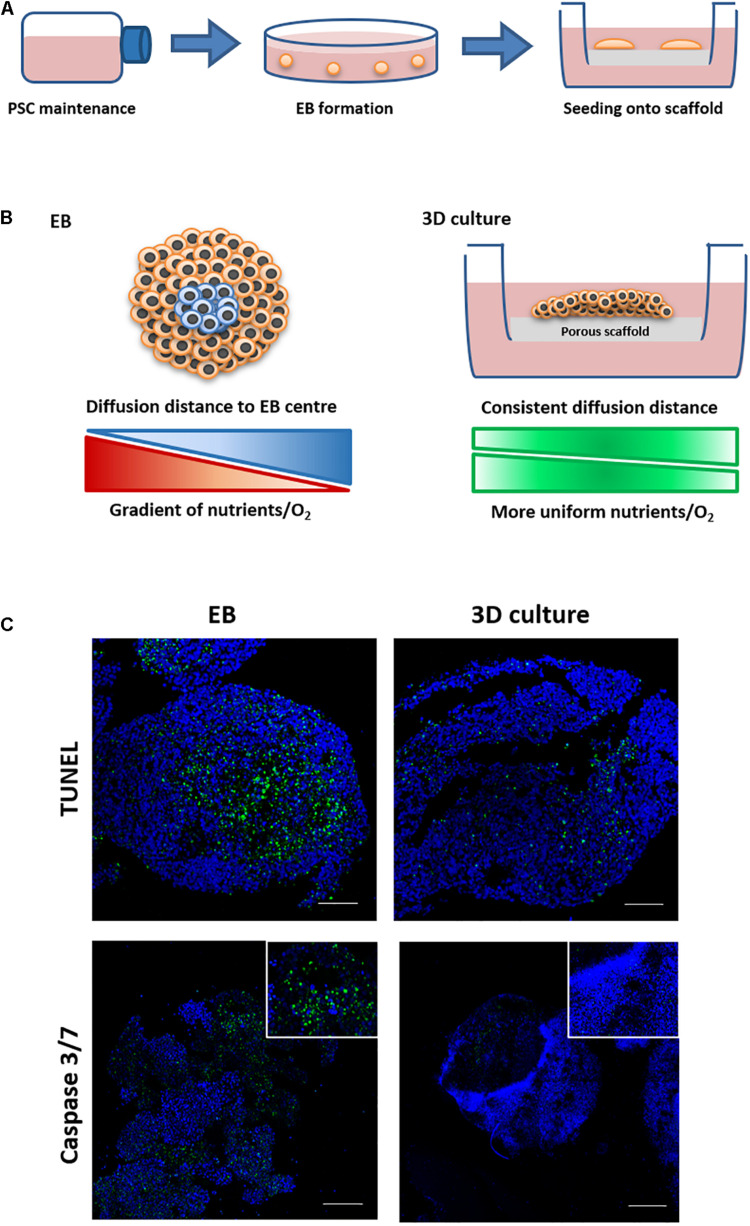
Schematic and rationale underpinning the development of new 3D culture method and its demonstration to enhance cell viability during the formation of tissue-like structures. **(A)** PSCs were routinely maintained in flasks and then used to form embryoid bodies (EBs) in low adherence dishes. EBs were transferred to Alvetex^®^ Polaris porous scaffold membranes and cultured for prolonged periods of time. **(B)** EBs cultured on the scaffold undergo a shape change forming flattened thinner 3D structures, reducing diffusion distances and allowing for more even distribution of nutrients and oxygen. This strategy allowed for the successful long-term maintenance of differentiating cells and the maturation of tissue structures similar to those identified in teratomas. **(C)** EBs were maintained in suspension culture for 14 days before being transferred to porous membranes or being maintained as spheroids in suspension conditions for a further 7 days. Cell death was assessed using two staining methods on live and fixed samples. Live samples were stained using a Caspase 3/7 stain. TUNEL staining was completed on fixed samples which had been paraffin embedded and sectioned. Both staining methods showed evidence of cell death in more central regions inside the spherical EBs maintained in suspension. There was minimal/no evidence of cell death when EBs were transferred onto the porous membranes and maintained for the same period of time as 3D cultures, as can be seen from the reduced staining for Caspase 3/7 and TUNEL. *n* = 3 independent experiments. Scale bars: 100 μm.

The purpose of this study was to develop a novel *in vitro* culture method which could be used to form more differentiated tissues similar to those observed in teratoma xenografts. This was achieved by the development of new technology enabling prolonged viability of PSC derived EBs permitting increased time for more extensive cell differentiation. EB formation is a common technique used to study basics aspects of human tissue differentiation and development. However, the method is limited due to the formation of a central core within the spheroid that displays poor viability and cell death as the EB increases in size. To overcome this issue and extend cell viability, a two-step culture method was developed, whereby PSC derived spherical EBs were subsequently seeded and maintained as flattened 3D “discs” of cells on the surface of the porous polystyrene membranes as shown in Figure 2A. We hypothesise that this transformation in EB shape results in the reduction of diffusion distances within the EB, leading to a more uniform distribution of nutrients and oxygen across its structure. This in turn should enhance cell viability and provide the potential for prolonged culture surpassing that which is normally feasible using spontaneously differentiating EBs maintained as spheroids in suspension culture (Figure 2B).

### Optimisation Steps Using Human Embryonal Carcinoma Stem Cells Demonstrate Proof of Concept With Improvements in Viability in 3D Membrane Cultures Compared to EB Spheroids

In a series of proof-of-concept studies, EBs formed from the EC stem cell line TERA2.cl.SP12 were seeded onto 200-micron polystyrene membranes to analyse cell viability. EC cells were used as they successfully form EBs and teratocarcinomas, embodying the properties of PSCs, but have simpler maintenance requirements to optimise the experimental technique. Time matched TERA2.cl.SP12 derived EB spheroids and EB derived 3D membrane cultures from the same starting population were analysed to assess differences in cell viability following 14 days in suspension, and a further 7 days either in suspension or cultured on the surface of a porous membrane. As shown in Figure 2C, EBs maintained in suspension showed evidence of positive staining for cell death in the centre when subject to TUNEL staining to detect the double stranded DNA breaks associated with the process of cell mortality. The 3D membrane cultures showed little to no evidence of positive TUNEL staining, indicating a lack of cell death when EBs are maintained on the porous scaffold. The two sample types were also analysed using a live caspase 3/7 stain (Figure 2C) and again evidence of positive staining was detected in the EB spheroid samples only, with the 3D membrane cultures maintained on the scaffold showing little indication of positive staining, further confirming their increased cell viability. Measurements of the diameter/thickness of the suspended EBs and 3D membrane cultures showed a notable difference. Feret’s diameter measurements of EBs gave a mean value of 529 μm (*n* = 10, SEM ± 15.67), and thickness measurements of 3D membrane cultures gave a mean thickness of 401 μm (*n* = 10, SEM ± 10.84). The average diffusion distance to the centre of each structure was calculated to be half of the Feret’s diameter/thickness, and was found to be 264 and 200 μm, respectively, for TERA2.cl.SP12 EBs and 3D membrane cultures.

### Maintenance of EC Stem Cell Derived EBs on 3D Scaffold Membranes *in vitro* Reproduces Aspects of TERA2.cl.SP12 Derived Teratocarcinomas *in vivo*

**FIGURE 3:**
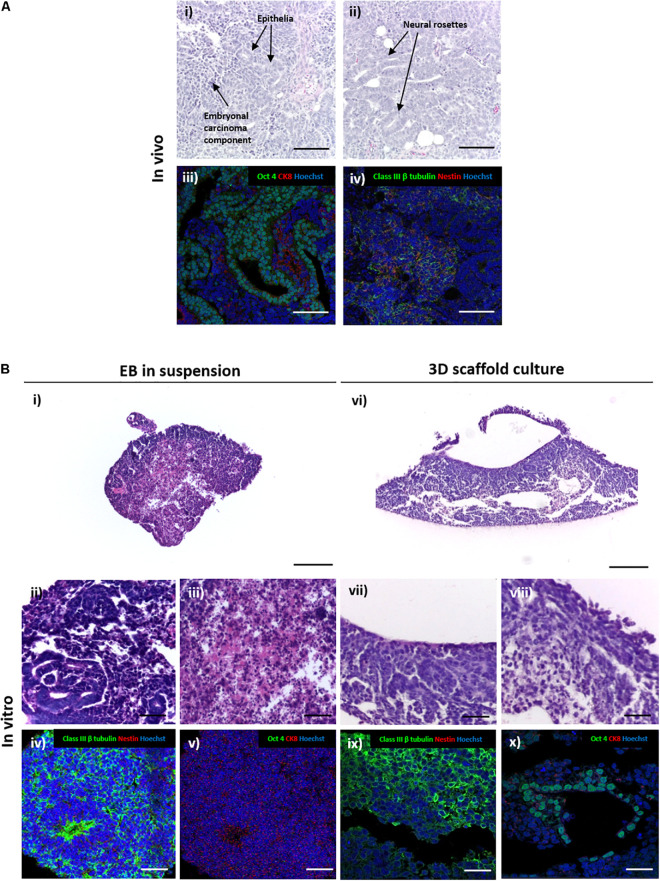
Comparison of the differentiation of embryonal carcinoma stem cells to form structures in teratocarcinoma xenografts *in vivo* and 3D cultures *in vitro.* **(A)** H&E staining in panel **(i,ii)** of TERA2.cl.SP12 derived teratocarcinoma xenografts revealed the heterogeneous nature of the tissues and structures within the tumour. Differentiated teratoma components typically consisted mainly of ectoderm derived structures, such as neural rosettes and epithelia in panel **(i,ii)**. The tumour also contained central regions of undifferentiated embryonal carcinoma cells which persisted in the xenograft in panel **(iii,iv)**. Immunofluorescent staining of neural markers Nestin and Class III β tubulin highlighted the neural tissues in panel **(ii)**, cytokeratin 8 stained the epithelia and the pluripotency marker Oct4 identified the undifferentiated embryonal carcinoma stem cells in panel **(iv)**. Representative structures shown. Scale bars: 200 μm. **(B)** Sections of TERA2.cl.SP12 cell derived EBs grown as suspended 3D spheroids in panel **(i–v)** or maintained on porous membranes in panel **(vi–x)**. H&E stained images of transverse sections of spherical EBs showed some evidence of differentiation in panel **(ii)**, as well as tissue degradation and cell death in central regions in panel **(iii)**. Immunostaining highlighted prominent neural differentiation in panel **(iv)**, but a distinct lack of Oct 4 positive embryonal carcinoma areas in panel **(v)**. By contrast, EBs maintained on the surface of porous membranes in panel **(vi–x)**, contained both the differentiated teratoma in panel **(vii)** and undifferentiated embryonal carcinoma in panel **(viii)** aspects of the teratocarcinoma, with evidence of neural differentiation in panel **(ix)**, particularly neuroepithelia, and Oct 4 positive pluripotent areas in panel **(x)**. Representative structures shown. Scale bars: **(Bi,vi)** 200 μm, all others 50 μm.

When used in the teratoma assay, EC stem cells form a teratocarcinoma xenograft tumour that contains both the differentiated teratoma and an undifferentiated EC component (Figure 3A). Histological analysis reveals the heterogeneous nature of the tumours with evidence of differentiated ectodermal tissue structures (TERA2.cl.SP12 EC stem cells show a preference for neural differentiation) and regions of distinctive rounded EC stem cells. Immunostaining for specific markers provides confirmation of the mixed nature of the teratocarcinoma: neural tissues were identified by positive staining for mature pan neuronal marker Class III β tubulin and early marker Nestin (Dhara and Stice, 2008; Nestor et al., 2013), epithelial structures were identified by positive staining for cytoskeletal marker cytokeratin 8, and regions of undifferentiated stem cells were detected using the pluripotency associated transcription factor Oct4 (Figure 3A).

Spherical EBs derived from TERA2.cl.SP12 cells maintained in suspension culture also showed evidence of differentiation with neural structures histologically visible that were confirmed by positive staining for neuronal marker Class III β tubulin (Figure 3B). However, positive staining for epithelial cytokeratin 8 and pluripotency marker Oct4 were not detected in suspension EBs indicating that the undifferentiated EC component was not maintained. Lower magnification images also showed degradation of the central regions of the spherical EBs indicating cell death resulting in a disorganised and fragmented structure. This area of cell necrosis correlated with the central region of proliferative EC stem cells that were no longer evident in the EB.

By contrast, the analysis of the EBs that had formed flattened 3D aggregates of cells maintained on porous scaffold membranes showed the formation of a more heterogenous tissue structure with no visible evidence of cell death (Figure 3B). Closer inspection identified differentiated neural and epithelial structures of teratoma, and areas resembling undifferentiated EC. Immunostaining confirmed the identity of these tissue structures with positive staining for Class III β tubulin and Oct4, respectively. The presence of differentiated neural tissues and undifferentiated EC stem cells indicates that the morphology of the 3D cultures maintained on the porous scaffold membranes more closely represent the structure of TERA2.cl.SP12 derived teratocarcinomas *in vivo*.

### Formation of Structures Similar to Those Observed in Teratomas Achieved by Differentiating Murine ES Cells for Up to 35 Days on Scaffold Membranes *in vitro*

**FIGURE 4:**
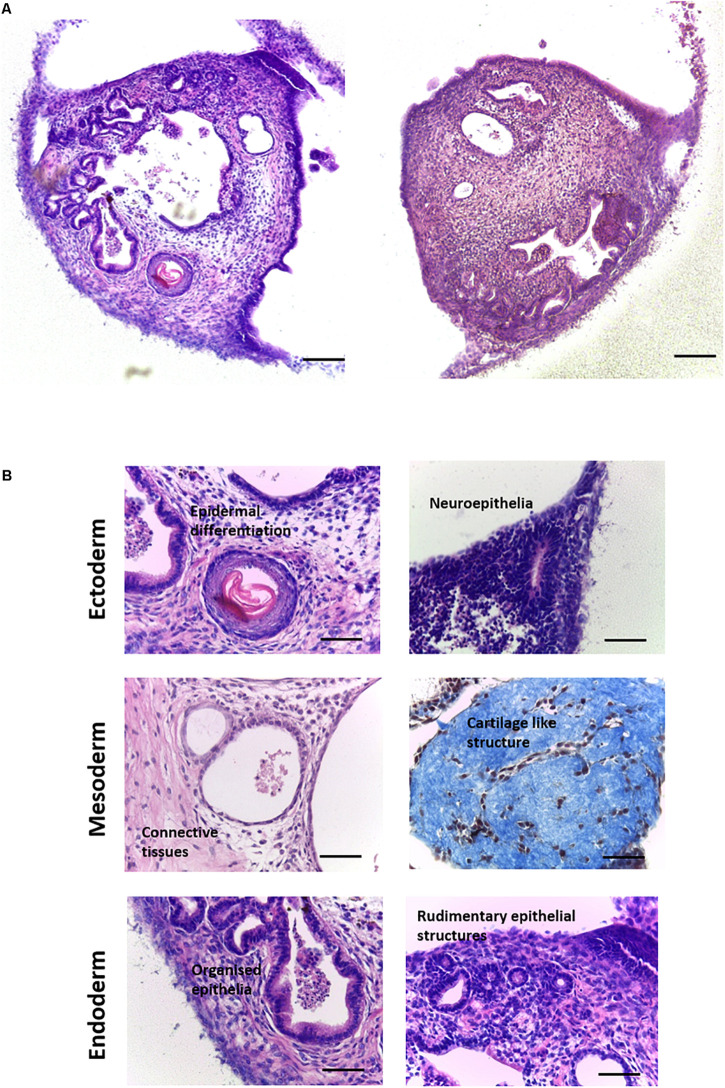
Formation of teratoma tissues derived from differentiating murine ES cells for up to 35 days on porous membranes *in vitro*: Histological analysis of H&E stained specimens derived from the CGR8 murine embryonic stem cell lineage identified a variety of differentiated structures within the 3D cultures. **(A)** Examples of culture heterogeneity in longitudinal cross sections viewed from above the membrane at low magnification showed a wide variety of tissue types, resembling the typical structure of a teratoma xenograft *in vivo*. **(B)** Further analysis at higher magnification revealed the detailed morphologies of tissue derivatives, with evidence of differentiation from all three germ layers: Ectoderm–epidermal and neural differentiation; Mesoderm–connective tissue, with mature cultures showing evidence of the formation of dense condensations of connective tissues, reminiscent of rudimentary cartilage (Masson’s trichrome stain); Endoderm: A variety of epithelial structures were identified, such as primitive gut epithelium, with evidence also of rudimentary kidney tubule formation. Representative structures shown. Scale bars: **(A)** 100 μm and **(B)** 50 μm.

Human EC cells were useful for undertaking the initial proof of concept and optimisation studies of this new method to support the differentiation of PSCs *in vitro*. Subsequently we used the murine embryonic stem cell line CGR8 to further test the technology. CGR8 derived EBs were seeded onto porous scaffold membranes and maintained for 14–35 days. Low magnification inspection of the differentiating cells showed that the 3D cultures contained a variety of recognisable, complex tissue structures, resembling the haphazard arrangement found in a teratoma (Figure 4A). Higher magnification imaging highlighted multiple structures representative of all three primary germ layers, each with distinct tissue morphology (Figure 4B). These included: ectoderm derived epidermal and neural differentiation; mesoderm derived connective tissues containing fibroblasts as well as structures reminiscent of rudimentary cartilage in more mature cultures; and a variety of endoderm derived epithelial structures including rudimentary intestinal and renal structures, with evidence of polarisation and primitive organisation.

**FIGURE 5:**
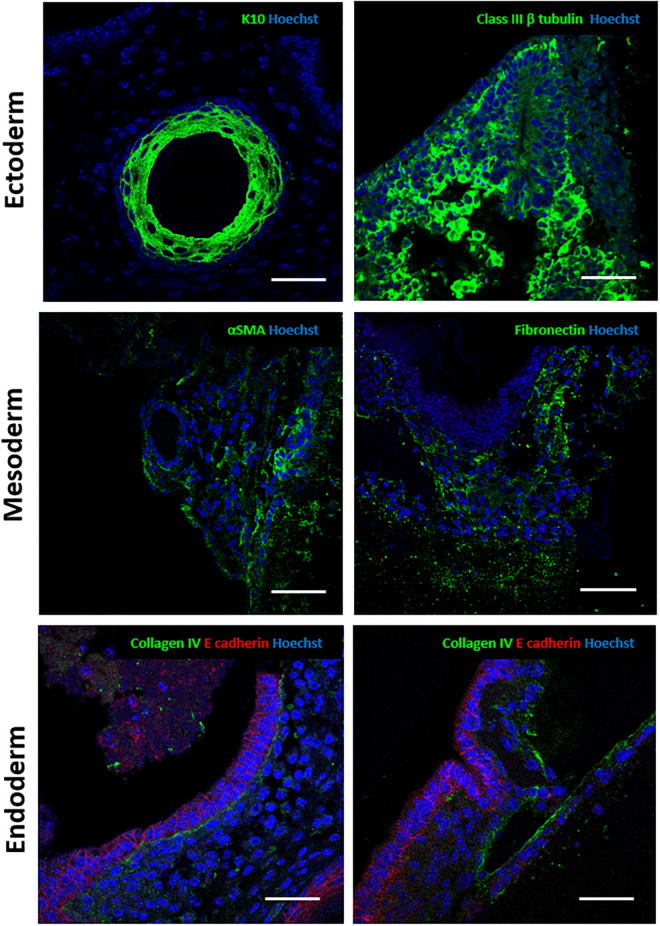
Immunocytochemical analysis of murine teratoma structures within the 3D cultures provides further validation of tissues present: Antibody staining against a number of germ layer markers was performed to further identify the tissues in the teratoma model. Ectoderm: epidermal structures stained positive for cytokeratin 10, a marker of epidermal differentiation. Positive staining for Class III β tubulin, a pan-neuronal marker, confirmed the presence of neural differentiation. Mesoderm: alpha smooth muscle actin positive staining indicated the presence of smooth muscle tissues. Fibronectin deposition indicated the presence of fibroblasts, a mesoderm derived cell type able to synthesise and deposit extracellular matrix and connective tissues. Endoderm: Various epithelial structures identified stained positive for E-cadherin, a junctional protein found in epithelial surfaces. Collagen type IV expression was confirmed in the basement membrane in some of these structures. Representative structures shown. Scale bars: 100 μm (Ectoderm and Mesoderm) and 50 μm (Endoderm).

Immunostaining was performed to further confirm the identity of the tissue structures observed (Figure 5). For ectoderm, epidermal differentiated tissue stained positive for early keratinocyte differentiation marker keratin 10 (Guenou et al., 2009). Neural tissues expressed the pan neuronal marker Class III β tubulin (Dhara and Stice, 2008; Nestor et al., 2013). Alpha smooth muscle actin stained positively in the 3D cultures and was used to identify certain mesodermal derivatives indicating the presence of myofibroblasts (Nestor et al., 2013). Expression of fibronectin, an extracellular matrix component, confirmed the presence of ECM secreting fibroblast populations (Kim et al., 2018). Due to the wide range of tissues derived from the endoderm, generic markers were used to further characterise various epithelial structures. E-cadherin, an epithelial cell junctional protein (Negoro et al., 2018), and Collagen type IV, an integral basement membrane protein found in organised differentiated tissues (Yurchenco and Patton, 2009; Sekiguchi and Yamada, 2018), are often expressed together in organised epithelial surfaces. Endoderm derived epithelial structures within the 3D cultures showed a range of morphologies: all had clear E-cadherin staining, outlining the organised nature of the cells within tissue rudiments and a Collagen type IV basement membrane, indicating organisation and polarisation.

### Directing Cell Differentiation Using Exogenous Morphogens Towards Tissues Associated With Particular Germ Layers

**FIGURE 6:**
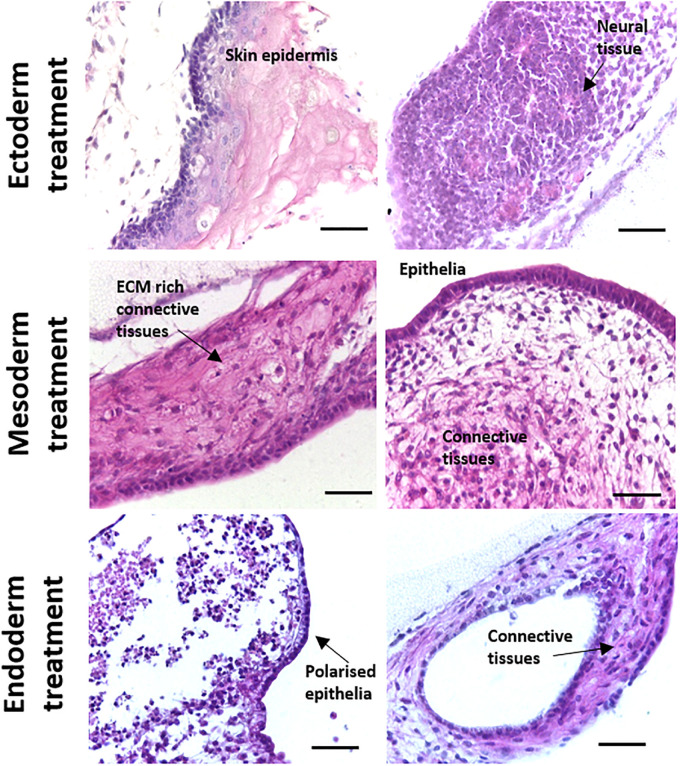
Directed differentiation of murine ES cells using combinations of alternative morphogens results in changes to teratoma structure towards corresponding primary germ layer. Cocktails of various morphogens were used to treat differentiating CGR8 murine PSCs to demonstrate that direction of cell and tissue differentiation could be manipulated when using this 3D culture *in vitro* technique. Histological analysis of teratoma tissue of H&E stained samples treated with different morphogens showed more substantial development of tissues associated with the respective germ layer. For example, samples directed towards ectoderm showed significant amounts of neural differentiation and formation of stratified skin epithelia. In contrast, cultures directed towards mesoderm and endoderm possessed significantly less ectodermal derivatives and more evidence of specialised epithelia and connective tissues. Scale bars: 100 μm (Ectoderm and Mesoderm) and 50 μm (Endoderm).

While the spontaneous differentiation of PSCs can provide valuable information regarding their general potency, directed differentiation experiments are particularly useful for studying mechanisms involved in the early stages of human tissue development. To test whether the differentiation of tissue structures could be directed preferentially towards certain germ layers, CGR8 EBs were treated with a combination of morphogens at the Aggrewell stage of the method. Morphogen combinations were taken from a recent International Stem Cell Initiative study (Allison et al., 2018) and were as follows: Ectoderm–10 μM dorsomorphin, 10 μM SB431542 and 100 ng/mL bFGF, Mesoderm–20 ng/mL Activin-A, 20 ng/mL BMP4 and Endoderm–100 ng/mL Activin-A, 1 ng/mL BMP4. Following treatment, EBs were maintained as previously described and seeded onto porous scaffolds. In general, there was a shift in the differentiated structures found in the tissues formed on the scaffold membranes towards the selected germ layer (Figure 6). EBs treated with ectoderm inducing morphogens showed an increase in the presence of neural and stratified epidermal structures. Similarly, simple epithelia and various connective tissues were more frequently observed in the EBs treated with a mesoderm inducing morphogen combination. Epithelia and connective tissues also constituted the main structures identified in the endoderm inducing morphogen combination, although there was some evidence of increased structural complexity with numerous examples of polarised epithelia present. It was also notable that there were significantly less ectodermal derivatives observed in samples treated with morphogens directing differentiation towards mesoderm and endoderm.

### Human Embryonic Stem Cells Also Form Complex Differentiated Structures When Maintained on Scaffold Membranes *in vitro*

**FIGURE 7:**
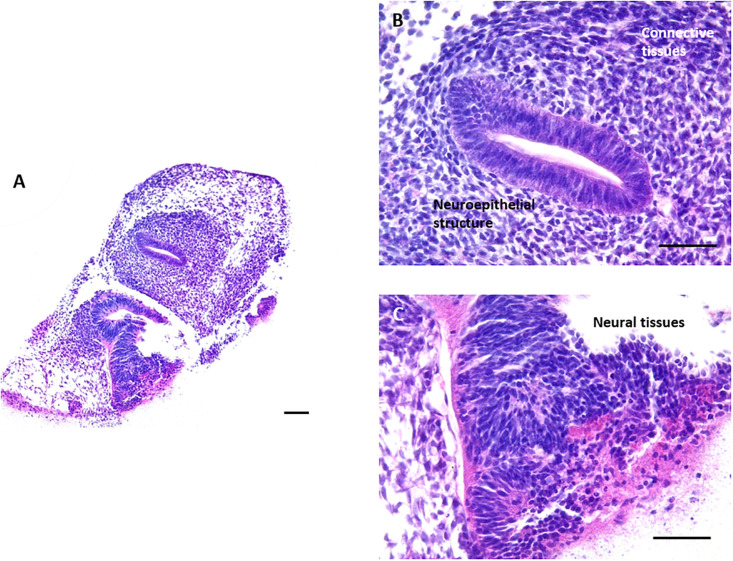
Formation of teratoma tissues derived from differentiating human ES cells for 18 days *in vitro*: Histological analysis of H&E stained samples derived from human embryonic stem cell line H9 demonstrated that complex structures can also be obtained from the culture of human EBs on the surface of the porous membrane. When allowed to spontaneously differentiate, human ES cell derived 3D cultures differentiated towards the default lineage of neuroectoderm, as can be observed from the numerous neuroepithelial and neural structures present within the 3D culture. Areas of mesodermal differentiation are also present in the surrounding connective tissues. Representative images shown. Scale bars: 100 μm in panel **(A)** and 50 μm in panel **(B,C)**.

In a final proof of principle experiment, the assessment of the ability of hESCs to differentiate and form complex 3D tissue structures was also confirmed using the technology developed as described herein. EBs were formed using the well characterised hESC line H9 (Thomson et al., 1998) and cultured on the surface of the porous scaffold for 18 days. Human stem cell derived 3D cultures maintained on porous scaffold membranes contained a variety of tissue structures, mainly ectoderm and mesoderm derived (Figure 7). Neuroepithelia and neural tissues, as a result of default differentiation along the ectoderm route, were frequently observed in the 3D cultures, often with surrounding masses of mesoderm derived connective tissues.

### The Novel *in vitro* Method Offers a Number of Advantages Over the Established *in vivo* Method

**FIGURE 8:**
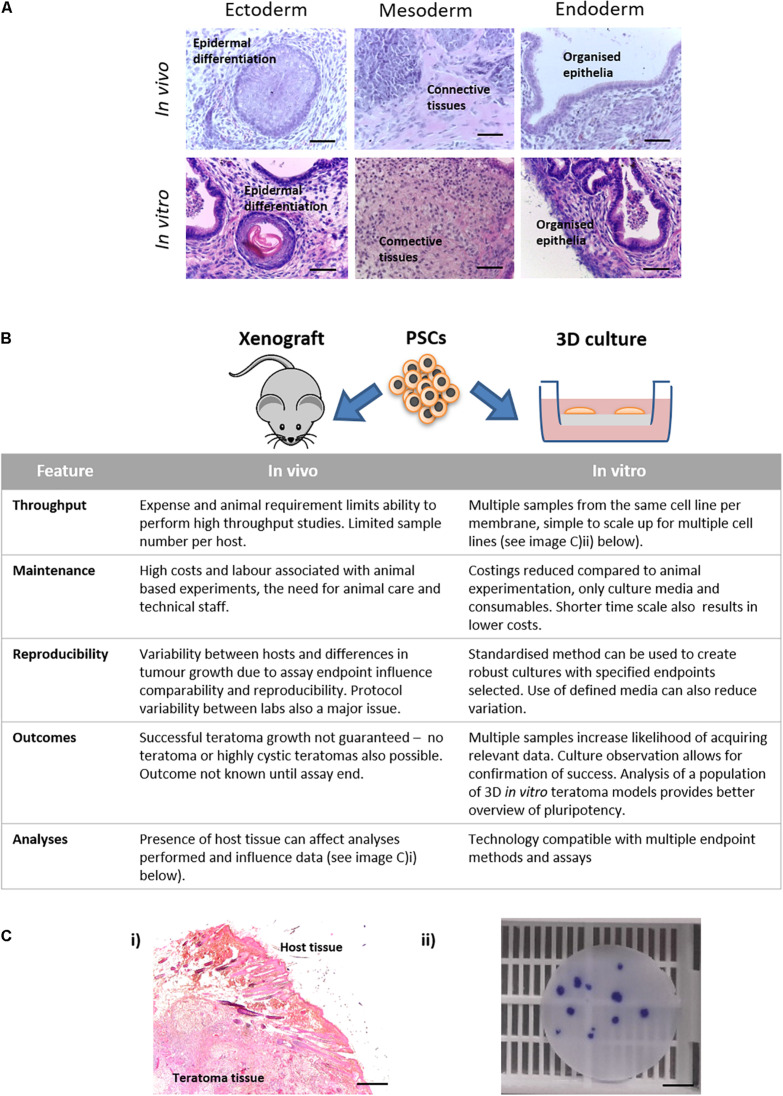
Features of *in vivo* and *in vitro* methods for producing teratoma tissues derived from PSCs: **(A)** Histological analysis of teratoma tissue of H&E stained samples derived from xenografts *in vivo* and 3D cultures *in vitro* formed using the same lineage of CGR8 murine embryonic stem cells showed evidence of differentiation from all three germ layers. Scale bars: 100 μm. **(B)** Features of the methods of producing teratomas either by the *in vivo* xenograft approach or by long term maintenance *in vitro* using the 3D culture technique as described herein, were compared. The *in vitro* platform offers a number of advantages including: the ability to undertake high throughput studies, both with the same cell population and with multiple cell lines; opportunities to direct differentiation *in vitro* by the addition of exogenous stimuli (e.g., morphogens); improved reproducibility; and reduced associated costs. **(Ci)** Formation of teratoma tissues *in vitro* avoids implantation and xenografts *in vivo* thus removing host/graft interactions. **(ii)** From a single porous scaffold, multiple independent 3D teratoma models can be produced for the same cell line. Image shows a 15 mm diameter membrane disc stained with Crystal Violet to identify individual EB derived structures. Scale bars: **(i)** 200 μm and **(ii)** 500 μm.

Up to this point, this article has explored the capabilities of this novel model using three different PSC populations, assessing their ability to form complex differentiated structures resembling teratoma xenografts. Comparing spontaneously differentiated CGR8 EBs maintained on the porous scaffold with teratoma samples created using the same cell population shows similar differentiated structures from each germ layer can be found within each sample type (Figure 8A). The similarity between these samples indicates the ability of this method to achieve complex, recognisable differentiated tissues which could be used to study human development and assess the pluripotency of human PSCs. As well as the similarity of the tissues produced, the technical aspects of the teratoma assay should also be considered and compared to the *in vitro* method. Due to the nature of the teratoma assay, the *in vitro* method is advantageous in a number of ways, including throughput and reproducibility (Figure 8B, expanded further in the “Discussion” section).

## Discussion

Currently, there are a variety of techniques available to study the differentiation of human tissues. These methods vary in their scope and depth, but are limited in a number of ways, whether that be technically, logistically or ethically. *In vitro* methods offer controllability, reproducibility and can recapitulate some aspects of these developmental processes, but often these techniques cannot achieve the tissue complexity and maturity observed when using *in vivo* methods. The teratoma assay is of particular significance, as the formation of highly complex, recognisable, mature differentiated tissues from all three germ layers can be achieved using PSCs (Figure 1). Yet, the technique is limited due to its use of animals, labour intensive nature and significant protocol variation between laboratories. In this study, we aimed to recapitulate aspects of the tissue complexity and maturity observed in the teratoma assay in the *in vitro* setting using a combination of EBs and subsequent maturation on a porous scaffold membrane.

Embryoid bodies have long been used to study embryogenesis, tissue differentiation and cellular pluripotency (Sheridan et al., 2012), since they possess some similarities to the early embryo (Martin et al., 1977). However, issues with EB viability can prevent their usage in longer term studies due to the formation of a core of cell death within the centre of the structures as they increase in size (Gothard et al., 2009; Son et al., 2011; Wu et al., 2014; Giandomenico et al., 2019). This is understood to be as a result of the formation of increasing nutrient and oxygen gradients across the EB (see Figure 2), leading to cell death as well as influencing the differentiation trajectory of EBs (Gassmann et al., 1996; Van Winkle et al., 2012). To improve EB viability and allow for long term cell differentiation and maturation, EBs were seeded onto a porous polystyrene scaffold in order to facilitate a shape transformation and create a microenvironment with a more uniform distribution of nutrients and oxygen. Similar strategies are used in *ex vivo* organotypic slice culture and to extend the differentiation, complexity, and longevity of neurospheres (Nestor et al., 2013; Giandomenico et al., 2019, 2021); indeed, the use of complex 3D culture strategies combining biomaterial scaffolds with PSCs in order to direct and enhance their differentiation has expanded significantly over the last decade (Willerth and Sakiyama-Elbert, 2019). Initial studies were carried out using a well characterised EC cell line (Przyborski, 2001; Clarke et al., 2017), due to shared characteristics with ES cell populations (Andrews et al., 2005) but simpler culture requirements, allowing for large numbers of EBs to be generated during method optimisation. To assess the impact of seeding EBs onto the scaffold on overall viability, cell death within the structures was analysed using a caspase 3/7 dye [upstream in the apoptotic cascade (Walsh et al., 2008)] and TUNEL staining (DNA breaks are a marker of later stages of apoptosis). Staining for both caspase 3/7 and TUNEL was found to be substantially lower/undetectable in the EBs cultured on the porous membrane when compared to those maintained as spheroids in suspension. These data indicate an improved cell viability, correlating with previous studies which undertook similar procedures to improve the local cellular microenvironment (Giandomenico et al., 2019, 2021).

The overall aim of this study was to recreate a technique that facilitates improved 3D differentiation of PSC derived tissue structures, and to enable a higher degree of tissue complexity *in vitro* with some similarity to the morphology observed in teratoma xenografts *in vivo*. Our experiments also focused on characterisation of the tissue structures in the *in vitro* model and in comparison, to *in vivo* samples. The typical architecture of a teratocarcinoma xenograft formed from the TERA2.cl.SP12 EC lineage, consists of differentiated teratoma structures mostly ectodermal in nature and an undifferentiated EC component typically located in more central locations. Analysis of TERA2.cl.SP12 derived spherical EBs showed the presence of a central necrotic region consistent with other previous studies (Gothard et al., 2009; Son et al., 2011; Wu et al., 2014). This coincided with the absence of undifferentiated EC stem cell component within the structure, as evidenced by the lack of staining for pluripotency marker Oct4. It is likely that as the spherical EBs increase in diameter, poor diffusion into more central regions is unable to sustain the growth of the proliferative metabolically active EC component hence its absence in these cultures. In contrast, there is little evidence of cell death in EBs that transformed their shape into more flattened structures when maintained on the porous membrane scaffold where diffusion distances to central regions were reduced. Measurement of the dimensions of the relative cultures further confirmed the hypothesised differences in diffusion distance, with that observed in the 3D membrane culture around 70% of the distance measured in the suspended EBs. Importantly, the diffusion distance to central regions of the 3D membrane cultures is around the tissue diffusion limit characteristic of oxygen in tissues, which is taken to be approximately 200 μm at most (Carmeliet and Jain, 2000; Rouwkema et al., 2009). Future studies utilising oxygen sensitive chemical probes as has been employed in other spheroid based systems (Raza et al., 2017) or assessing the expression of the oxygen regulated transcription factor Hypoxia Inducible Factor 1-alpha (HIF1α) would provide further information as to the oxygen gradients across the differentiated structures. Under the 3D growth conditions on the porous membranes, TERA2.cl.SP12 cells formed heterogeneous structures containing both differentiated teratoma (neural tissue positive for Class III β tubulin) and undifferentiated EC (Oct4 positive) components. These data highlighted the ability of the technique to more faithfully reproduce the 3D structure of a teratocarcinoma *in vitro* with closer similarity to the structure of a xenograft derived *in vivo*. Moreover, these initial results demonstrated proof of concept for further studies involving more relevant PSC lineages.

Murine PSC lines are an accepted test population for testing the development of new techniques due to their relatively simple culture requirements and similarities to human PSC populations (Schnerch et al., 2010). The common murine embryonic stem cell line CGR8 was selected for use in experiments to further characterise the capabilities of the 3D *in vitro* model described herein. The culture of differentiating CGR8 PSCs on the porous scaffold membrane resulted in the formation of a wide range of tissue structures even after only 14 days. Many had distinct morphology and maturity with evidence of differentiation of all three primary germ layers, fulfilling this single yet critical success criterion of the teratoma xenograft assay (Brivanlou et al., 2003; Hentze et al., 2009).

The disorganised yet mature nature of the 3D cultures maintained on the porous membranes was also highly reminiscent of a teratoma that typically forms spontaneously. In an attempt to provide more direction to such differentiation, our experiments confirmed that it was possible to guide tissue formation within the 3D structures towards specific germ layers through the application of known exogenous morphogens found in developing embryonic tissues. While such differentiation experiments still showed some variation, there was a clear and noticeable shift in the formation of the tissue structures produced towards the corresponding germ layer. Our initial directed differentiation experiments were relatively simple in nature, involving a single application of morphogens at the beginning of EB formation, but were sufficient to provide preliminary data showing that this PSC tissue differentiation system can be further controlled. While the study on which these morphogen experiments was based also scored the strength of differentiation of the PSC lines in terms of propensity (spontaneous differentiation) and potential (directed differentiation) to form each germ layer in the EBs (Allison et al., 2018), the qualitative nature of the analyses in this study prevents this. Qualitative analyses such as these are more subjective by nature and coupled with the variable and haphazard nature of the EBs/teratomas, these qualitative analyses can only really be used to ascertain trends in differentiation rather than directly comparing the strength of differentiation. While some attempts to more quantitatively assess the histology of teratomas have been made (Oh et al., 2009; Bhagavatula et al., 2010), ultimately quantitative gene expression analyses will provide more objective data as to the strength of differentiation. These simple morphogen studies lay the foundations for more nuanced differentiation experiments, that could involve exposing the 3D cultures to a series of alternative morphogens at different times and concentrations to simulate developmental processes. Accordingly, these approaches will be particularly valuable in the study of embryonic tissues using human pluripotent embryonic stem cells. Herein we demonstrated that our 3D culture technique was also compatible with the hESC lineage H9. Initial results showed that H9 derived EBs can also be successfully maintained as 3D cultures on the porous scaffold membranes and showed the ability to form differentiated structures representative of multiple germ layers, notably the default neuroectoderm lineage. The lack of endodermal derivatives in the H9 samples is noted, and may simply be due to the fact that the default differentiation pathway for hESCs is neuroectoderm (Vallier et al., 2004), the inherent differences in lineage preference of human PSCs (Osafune et al., 2008) or that more complex media are required to achieve endodermal differentiation (Harkness et al., 2019). In summary, these results pave the way for further experiments demonstrating the capabilities of this novel 3D cell culture technique: focusing on longer term differentiation to achieve more mature and complex tissue structures from all three germ layers *in vitro;* and steering their differentiation towards more specific derivatives. Collectively these approaches will assist in the study of developmental processes, particularly during the formation of rudimentary tissues in man.

Another important feature of this technology is highlighted in Figure 8, which presents evidence to justify the use of our novel *in vitro* method as a possible replacement for the teratoma xenograft assay *in vivo*. The teratoma assay is an established surrogate technique used to assess the functional pluripotency of human PSCs (Bouma et al., 2017), and has long been considered the “gold standard” method, primarily due to the fact that more stringent methods such as germline transmission and tetraploid complementation are unavailable for use with human cells due to the formation of chimera species (Aoi, 2016). While the teratoma assay is able to provide “reliable and comprehensive” proof of the pluripotency (Zhang et al., 2008), the limitations of the assay and need for change are very well recognised within the field (Müller et al., 2010; Gropp et al., 2012; Buta et al., 2013; Bouma et al., 2017). At the very least, the assay requires standardisation and the setting of minimum reporting standards to allow for useful comparisons regarding cellular potency (Müller et al., 2010), but ideally, replacement with a more reproducible technique that does not use animals would be most desirable. As highlighted in Figure 8, our *in vitro* approach offers a number of advantages over the *in vivo* assay, including the ability to increase sample number, both within the same cell line and with additional cell lines, but with overall reduced associated costs and maintenance requirements when compared to studies using animals. Variability and reproducibility are naturally improved when using the *in vitro* method through consistency of the culture environment and there is the opportunity to implement a standardised protocol more easily. This would allow for comparison between samples which would provide useful insights into the potency and behaviours of different PSC lineages, given their inherent variability (Cahan and Daley, 2013; Ortmann and Vallier, 2017). Most notably use a more rapid screening tool when assessing the potency of induced PSCs, which are often poorly characterised (Müller et al., 2010; Leha et al., 2016). When undertaking the *in vivo* xenograft assay, there is often the risk that a tumour may not form, or may be cystic in nature, and in the latter case this may not be realised until the tumour is excised, leading to a waste of valuable resources and time. This is not an issue with the *in vitro* assay, and the increased sample number significantly improves the likelihood of obtaining significant amounts of valuable data. The compatibility of the *in vitro* method with a wide range of analytical techniques and the absence of host tissue which could interfere with data interpretation are additional advantages of the *in vitro* method, permitting more in-depth investigations into pluripotency and mechanisms of cellular differentiation.

This novel *in vitro* model represents a significant step forward in characterising the potency and differentiation capacity of PSCs in a more standardised, reproducible and animal free manner, enabling the formation of complex tissue structures resembling those found in the xenograft teratoma. However, the cellular microenvironment in this *in vitro* model is much simpler than that found in the xenograft microenvironment *in vivo*. A much wider range of microenvironmental factors influence behaviours and differentiation of the PSCs *in vivo*; these include vascularisation of the tumour, presence of circulating cytokines/morphogens, differences in ECM content/stiffness and differences in local oxygen tension, among many others. The potential to incorporate these additional cues and factors using advanced cell culture techniques provides an opportunity to further develop the model, with the ultimate aim of further improving the differentiation outcomes and the formation of increasingly complex tissues derived from PSCs *in vitro*.

## Conclusion

In this study, we have developed a novel *in vitro* technique utilising a porous membrane and 3D cell culture technology to prolong the viability of PSC derivatives and their maturation into tissue structures. This methodology has been developed and demonstrated using human EC cells, murine embryonic stem cells and hESCs, each capable of producing tissue rudiments comparable to those found in xenograft tumours derived from the respective cell population. Importantly, we have created an animal free alternative to the teratoma xenograft assay, overcoming many of the limitations and ethical concerns associated with this approach. We propose that this new technique can be used to study developmental mechanisms and investigate new strategies to generate tissues from stem cells, providing a foundation on which more complex work can be built to better understand the complexities of tissue differentiation.

## Data Availability Statement

The original contributions presented in the study are included in the article/supplementary material, further inquiries can be directed to the corresponding author.

## Ethics Statement

Ethical review and approval was not required for the animal study because all procedures were completed under licences (PPL60/4508 and PPL60/3579) and permission according to the guidelines of the Home Office, United Kingdom.

## Author Contributions

LS, AH, DO, RQ, and EK performed the experiments. LS and AH conducted the data analysis. LS, AH, and SP wrote and revised the manuscript. SP had oversight of the project and approved the manuscript for submission. All authors contributed to the article and approved the submitted version.

## Conflict of Interest

Author SP is affiliated with the company Reprocell Europe. The remaining authors declare that the research was conducted in the absence of any commercial or financial relationships that could be construed as a potential conflict of interest.
